# Implementing evidence-based eating disorder guidelines at a small metro hospital: current practice and staff perceptions of caring for eating disorder patients

**DOI:** 10.1186/s40337-023-00787-y

**Published:** 2023-04-20

**Authors:** Stacey L. O’Donnell, Nina J. L. Meloncelli

**Affiliations:** 1 Department of Nutrition and Dietetics, Caboolture Hospital, Caboolture, QLD Australia; 2 Metro North Health, Office of the Chief Allied Health Practitioner, Brisbane, QLD Australia

**Keywords:** Eating disorders, Implementation, Guidelines, Nutrition, Knowledge translation

## Abstract

**Background:**

The aim of this study was to understand current clinical practice, adherence to evidence-based guidelines, and the perceptions, knowledge and attitudes of the multidisciplinary team caring for inpatients with an eating disorder at a small metro hospital.

**Methods:**

This mixed methods study involved a retrospective audit of eating disorder patient care and a semi-qualitative staff survey. The audit was undertaken at a small metro hospital from 2018 to 2019. Documented practices were compared to state-wide best-practice guidelines. A staff survey was designed to understand health care professional’s knowledge and use of evidence-based practice guidelines, as well their perception of caring for this patient population and areas for improvement.

**Results:**

Twenty-three discrete admissions (18 individuals) were included in the audit. Findings highlighted several evidence-practice gaps including delayed nutrition initiation and inconsistent medical refeeding and management of refeeding risk. Survey themes (from 60 hospital staff) included: lack of confidence with providing eating disorder care; uncertainty about professional roles/responsibilities; and lack of clear processes/guidelines to inform clinical care.

**Conclusions:**

Gaps exist between evidence-based practice and eating disorder patient care. Staff lack confidence providing care to this patient group. These findings will allow for targeted implementation strategies to improve patient care and the uptake of research into practice.

## Abstract (summary)

Best practice guidelines for inpatient eating disorder (ED) care have not been consistently well implemented at our regional hospital. This research project aimed to address this problem in two phases. Phase one involved understanding: (1) current clinical practice and adherence to evidence based guidelines through a retrospective clinical audit and (2) the perceptions, barriers, enablers and attitudes of the multidisciplinary team providing ED care using a staff survey. The results of these activities highlighted areas for improvement and will be used to guide implementation strategies to align patient care with evidence based practice.

## Background

Eating disorders (ED) are severe, complex, and often debilitating mental health conditions, characterised by a severe and persistent disturbance in eating behaviour [1]. Approximately 1 in 20 Australians report having an eating disorder [2]. These disorders carry the highest mortality rate of all psychiatric illnesses [3]. Data suggests that approximately 10% of eating disorder patients are admitted to hospital with a life-threatening complication of their illness [1]. Early intervention is required to reduce the severity, duration and impact of the illness. It is estimated that only 1 in 10 people with eating disorders receive appropriate treatment [4], and variation to inpatient treatment occurs across Australia [5].

Given the complex medical and psychiatric needs of eating disorder patients, expert consensus recommends medical management should run in parallel with coordinated multidisciplinary team (MDT) care. Braude et al. (2020) examined the outcomes of 60 individual patients admitted for eating disorders [6]. The study described medical complications and outcomes pre- and post-implementation of multidisciplinary treatment guidelines. They found that following the implementation of MDT guidelines for eating disorder inpatient treatment, lower rates of refeeding electrolyte derangement were seen [6].

Individuals with eating disorders, including those who are medically compromised without presenting as underweight, are often at risk of not being admitted to hospital despite requiring urgent medical attention [7]. In a similar vein, severely ill individuals with an eating disorder may require urgent nutritional rehabilitation yet present without obvious medical instability [7]. Even when admitted, treatment can be inconsistent.

In 2017, updated state-wide guidelines were published by the Queensland Eating Disorder Service (QuEDS) [8], a publicly funded eating disorder treatment and consultation service in the third largest (by population) Australian state. The guidelines aim to assist clinicians to manage and address eating disorder risks by offering evidence-based indicators for admission to hospital and subsequent treatment recommendations for patients admitted to either acute medical or mental health wards [8]. The QuEDS guideline provides clear treatment recommendations for multidisciplinary teams to achieve the goals of inpatient ED treatment which are: medical stabilisation; prevention and treatment of refeeding syndrome, nutritional resuscitation and rehabilitation as well as safe discharge planning [8]. The guidelines are based on recommendations from the Royal Australian and New Zealand College of Psychiatrists as well as best available evidence relating to acute eating disorder care [9]. However, without a systematic approach to implementation, guidelines in isolation are rarely translated into practice.

Knowledge translation, commonly defined as “*the synthesis, dissemination, exchange and ethically sound application of knowledge to improve health”*, can assist with closing the evidence-practice gap [10]. It requires an understanding of the local and organisational context, and barriers and enablers to practice change to select strategies that can enhance the success of implementation [10]. Use of implementation theories, models and conceptual frameworks are recommended to improve knowledge translation and the uptake of evidence into practice [10]. In Queensland, the Allied Health—Translating Research Into Practice (AH-TRIP) initiative was developed to embed knowledge translation from research into Allied Health business as usual practices by building the capacity of the health practitioner workforce [11]. An opportunity to improve the management of eating disorder patients at a small metro, Queensland hospital by taking an AH-TRIP approach was presented. At the commencement of this project, there had been no hospital-wide implementation of the QuEDS or other local guidelines for the management of patients admitted for eating disorder treatment. The MDT was supportive of improving care and better understanding the evidence-practice gaps to guide change.

This dietitian clinician-led project, guided by the AH-TRIP process [11] included experienced facilitation and implementation project support. This two phase implementation project consisted of (i) understanding the local problem, mapping the evidence-practice gaps that existed, and undertaking a context assessment to assist with planning for implementation, and (ii) the implementation of strategies to improve eating disorder care and evaluate and any changes made. Here we report the outcomes of the first phase of the implementation project. The aim of this project was to understand the current practices, and the perceptions, and attitudes of the multidisciplinary team involved in the inpatient treatment of patients with an eating disorder. This information was used to develop an implementation plan to improve eating disorder treatment. The specific objectives were: (i) Examine the current practices relating to the medical and nutritional management of patients admitted for an eating disorder between 2018 and 2019: (ii) Understand how the management differs from the QuEDS guidelines; (iii) Describe staff perceptions and attitudes of managing patients with an eating disorder, including any barriers and enablers to guideline implementation; (iv) develop an implementation blueprint, including published implementation strategies designed to overcome reported barriers.

## Methods

This was a mixed methods study involving a retrospective clinical audit to understand current practices and the evidence-practice gap and a staff survey to understand staff perceptions of caring for eating disorder patients and barriers/enablers to guideline implementation.

The project was set in a 249 bed Queensland regional hospital which includes both medical and mental health inpatients. The hospital admits between 10 and 20 eating disorder patients annually either as direct admissions or interhouse transfers from other hospitals. This was a dietitian-initiated and led project, following recognition that taking a consistent approach to treatment and monitoring of adult eating disorder patients, in line with the state-wide guidelines, would provide opportunities for improved care, particularly relating to the commencement and monitoring of nutritional resuscitation and medical nutrition therapy.

### Retrospective audit of current practices and understanding the evidence-practice gap

Current practices were examined using a retrospective chart audit of routinely collected medical and administrative data. The patient group included in this study were medical or mental health inpatients who received care for an eating disorder at the hospital between 1st January 2018 and 30th December 2019. Patients were included if eating disorder management was indicated and undertaken at some stage during the admission, even if the eating disorder was not the primary reason for admission. Patients ≤ 16years or admitted to the paediatric ward were excluded. Patients who received a diagnosis of Avoidant Restrictive Food Intake Disorder were also excluded. Patients were identified through clinical recording coding obtained through hospital administration.

The hospital medical records of patients who met the study population criteria were audited and data was collected using a REDCap online collection tool developed specifically for this project. This 118 item tool was developed using the QuEDS guidelines [8] to understand the level of alignment between current practice and the guideline recommendations for the medical and nutritional management of eating disorder patients. Specifically, the audit was designed collect data on what was occurring in practice compared to the following QuEDS guideline recommendations [8]: indicators for admission and admission outcomes, initial assessment; medical management and monitoring; nutritional management, including insertion of nasogastric tube per recommendations; assessment and management of refeeding risk; patient observations in medical wards; admission and treatment to mental health wards; an achievement of appropriate weight goal recommendations. This was done to highlight the evidence-practice gap between what is recommended best-practice and what occurs in practice to highlight opportunities for improvement and to focus implementation efforts. Patient demographics, anthropometric data, length of stay, medical complications, biochemical measures and nutrition provision were also collected. All data were deidentified prior to analysis.

### Staff survey

To understand staff's knowledge and use of evidence-based practice guidelines, as well as their perception of caring for eating disorder patients, a staff survey was developed using the online survey tool, Microsoft Forms. Pre-existing survey tools were not identified through a literature search; however, a tool from Harken et al. [12] was adapted for the current project and reviewed by two senior clinicians for content and readability. The 10-item survey consisting of multiple choice, Likert scale and free text responses asked staff about: their profession; awareness of QuEDS guidelines, how likely they are to refer to the guidelines; whether they felt there were clear process to guide ED management and whether the staff member had a clear understanding of their role when caring for ED patients. The survey questions asked staff to report their level of confidence (where 1 was not at all confident to 10 very confident) caring for eating disorder patents and knowledge of evidence-based practices guidelines, as well as qualitatively (free text) reporting their perceptions on positive (enablers) and negative (barriers) aspects of eating disorder care provision. Finally, staff were asked to provide suggestion for improving care as a free text response.

The survey was distributed to the following staff working with eating disorders: dietitians, nursing staff working on acute medical wards and mental health wards, psychiatry consultants and their teams and medical consultants and their teams. Multiple methods to disseminate the survey were undertaken to gather the most representative sample of staff responses as possible including email invitation, paper copies, QR codes. The survey remained open for 4 weeks. Consent was implied through agreement to complete the survey.

#### The implementation process (developing an implementation blueprint)

Overall, the project was supported by an experienced ‘AH-TRIP’ facilitator, who was also an Accredited Practising Dietitian and clinician-researcher. Additional support was provided to the project lead through monthly AH-TRIP tele mentoring panel [13]. TRIP knowledge and capability for the project lead was incrementally gained through educational material on the AH-TRIP training platform [11] and skills were formed by the ‘doing’ of project activities. The AH-TRIP recommended steps to translating research into practice was used to guide the process [11]. The multi-step approach to planning for implementation (chart audits, mapping the evidence-practice gap and staff survey) were used to develop specific project activities to improve care. These objectives were then mapped to the Expert Recommendations for Implementing Change (ERIC) taxonomy to develop an implementation blueprint [14].

### Statistical analysis

Categorical variables were summarised using frequencies (%) and continuous variables using mean (SD). Non-normally distributed data were reported using median (interquartile range). Missing data accounted for less than 5% for any variable and data were not imputed. For the staff survey, a pragmatic thematic approach to examining free text responses was taken. The two authors independently grouped the responses into overarching themes to summarise reported enablers and barriers to ED patient care and any suggestions for improvements. Any disagreements in theming were settled via discussion. Multiple choice and Likert scale questions were reported descriptively. Respondent checking was not undertaken. Statistical analyses were carried out using IB SPSS Statistical Package 20.

## Results

Between 1 January 2018 and 30 December 2019, 23 discrete admissions were included from 18 individuals (Table 1). Eight admissions were represented by three individuals (two admissions for two patients, three admissions for one patient). Most patients (n = 20, 87%) were diagnosed with an eating disorder prior to admission and anorexia nervosa was the most common form of eating disorder diagnosis (n = 17, 74%) (Table 1). The average length of stay (LOS) was 21.7 ± 10.2 days, and most admissions (65%) were on a Friday, Saturday or Sunday (Table 1). LOS was increased when patients were admitted on a Friday, Saturday or Sunday (with the exception of one patient). There was no statistically significant difference to LOS if the patient was admitted via an inter-hospital transfer (IHT) or not (19.6 ± 10 vs. 23.3 ± 10.5, p 0.402).

**Table 1:** Patient demographics and admission details of inpatient eating disorders based on a retrospective clinical audit

Parameter	
Age, years (median + IQR)	22 (8)
Gender	
Male, *n* (%)	2 (9%)
Female, *n* (%)	20 (87%)
Indeterminate *n* (%)	1 (4%)
Body mass index on admission (median + IQR)	16.9 (3.8)
Eating disorder diagnosed prior to admission, *n* (%)	20 (87%)
Eating disorder diagnosis	
Anorexia Nervosa, *n* (%)	17 (74%)
OSFED, *n* (%)	4 (17%)
Atypical AN, *n* (%)	2 (9%)
Assessed in the emergency department, *n* (%)	10 (44%)
Average time in emergency department, hours (mean SD)	6.7 (2.5)
Admission details	
Direct admission, *n* (%)	13 (57%)
Interhospital transfer, *n* (%)	10 (44%)
Medical ward, *n* (%)	3 (13%)
Mental health ward, *n* (%)	16 (67%)
Both medical and mental health wards, *n* (%)	5 (21%)
Admission day	
Weekday (Monday to Thursday), *n* (%)	8 (35%)
Friday, *n* (%)	8 (35%)
Weekend (Saturday and Sunday), *n* (%)	7 (30%)
Length of stay, days mean (SD)	21.7 (10.2)
Prior admission for eating disorder treatment	
Past 6 months, *n* (%)	8 (35%)
Past 12 months, *n* (%)	10 (43%)
Required a treatment authority at any stage of admission, *n* (%)	16 (70%)

### Current practice and alignment with QuEDS guidelines

According to the QuEDS indicators for admission to adult inpatient beds [8], 65% (n = 15) of patients admitted during the audit time period met the criteria for a medical admission. Risk of refeeding (n = 12) and postural tachycardia > 20 beats per minute (n = 11) were the most common medical admission criteria met, followed by arrhythmia (n = 5), hypoglycaemia (n = 4) and electrolyte derangement (n = 4). During their admission 21% (n = 5) experienced electrocardiogram (ECG) changes (1 not measured), 65% (n = 15) experienced postural tachycardia > 20 bpm, and 56% experienced at least one episode of hypoglycaemia. 43% (n = 10) of patients did not have their blood glucose level taken on admission.

50% (n = 8) of patients admitted directly to the mental health ward met the QuEDS criteria [8] for a medical admission and five of those patients were assessed by the dietitian as at risk of refeeding. One patient was commenced on thiamine prior to feeding while the other patients did not received thiamine until day 2, 5 or 11. 75% (n = 5) of these patients experienced postural tachycardia during their admission and the same number also developed hypoglycaemia during their admission. No patients experienced an adverse event requiring transfer to a medical ward.

#### Nutritional management and complications and alignment with QuEDS guidelines

100% of patients were referred to the dietitian and 52% (n = 12) of patients saw a dietitian on day 0 or day 1 of their admission. An admission on Friday, Saturday or Sunday resulted in a delay to dietitian assessment due to lack of weekend dietetics services. Therefore, 56% did not see a dietitian for two or more days (Table 2). Less than one quarter of patients achieved a two body mass index (BMI) band increase as recommended by the QuEDS guidelines, prior to discharge (Table 2). However, all patients gained weight during their admission.

Of those (n = 14, 61%) who were recommended by the dietitian to receive enteral nutrition via a nasogastric tube (NGT), 71% (n = 10) were commenced on enteral nutrition. The dietitian initiated the enteral nutrition plan 100% of the time. 65% of patients were assessed by the dietitian as at risk of refeeding syndrome on admission (n = 15, Table 2) and those who were assessed as a refeeding risk were commenced on thiamine 100% of the time. 17% experienced mild to moderate electrolyte derangement during the refeeding period (Table 2). On average, it took 9.2 days for patients who were refed on the medical ward to achieve medical stability. The average LOS for these patients was 18.4 days (results not shown). There were no severe adverse events requiring escalation of care for any patient on the medical or mental health wards.

**Table 2:** Dietetic management, anthropometric data and electrolyte derangement of inpatient eating disorder admissions

Parameter	
Seen by dietitian within 24 h, *n* (%)	12 (52)
Not seen by dietitian within 48 h, *n* (%)	11 (48)
Total dietetic time per patient, hours mean (SD)	10.6 (6.7)
Dietetic time per patient per week, hours	3.1
Dietitian occasions of service, median (IQR)	6.5 (3)
Dietitian occasions of service per patient per week	2.2
Delay to dietetic review due to Friday, Saturday Sunday admission, *n* (%)	13 (56)
Weight gain (kg), mean (SD)	3.25 (3.2)
BMI change (kg/m2), mean (SD)	1.2 (1.1)
Two BMI band increase prior to discharge, *n* (%)	5 (21)
Days to appropriate feeding plan (NGT or meal plan), median (IQR)	2 (2)
Receiving appropriate feeding plan during admission, *n* (%)	21 (91)
Met goal feeding plan within 7 days of admission, *n* (%)	14 (61)
Requiring NGT, *n* (%)	14 (61)
Receiving NGT (% of those requiring), *n* (%)	10 (71)
Duration of NGT, *n* (%)	
< 1 week	3 (30%)
1–2 weeks	5 (50%)
3–5 weeks	2 (20%)
Assessed at risk of refeeding on admission, *n* (%)	15 (65%)
Received thiamine prior to feeding (% of those indicated), *n* (%)	6 (40%)
Hypoglycaemia on admission, *n* (% of those assessed), *n* (%)	2 (14%)
Hypoglycaemia during admission, *n* (%)	13 (54%)
Refeeding electrolyte derangement, *n* (%)	4 (17%)

#### Staff survey

Sixty staff responded to the survey comprising of 38 nurses, 10 medical officers, 9 dietitians and 3 psychiatrists (denominator unknown). 80% (n = 48) of staff surveyed were aware of the QuEDS guideline and 57% (n = 34) indicated that they were very likely to refer to the guideline to assist their clinical practice.

**Table 3:** Total and profession specific responses to survey questions

Profession	Dietitian (n = 9)	Nurse (n = 38)	Medical officer (n = 10)	Psychiatrist (n = 3)	Total (n = 60)
Aware of QuEDS guideline (Yes)	9 (100%)	27 (71%)	9 (90%)	3 (100%)	48 (80%)
Very likely to refer to QuEDS guideline	9 (100%)	18 (47%)	6 (60%)	1 (33%)	34 (57%)
Adequate local processes/ Guidelines (No)	6 (67%)	23 (61%)	6 (60%)	2 (67%)	37 (62%)
Clear understanding of roles (No)	1 (11%)	16 (42%)	3 (30%)	0 (0)	20 (33%)
Confidence providing care (Mean score on scale from 1 to 10 where 10 is highly confident)	6.6	6.2	6.5	7.3	6.3

Staff reported that they lacked confidence providing eating disorder care (Table 3). The average ranking for confidence in providing care for eating disorder patients was 6.3/10 (Table 3). More than 60% of staff do not believe that the hospital had clear processes guidelines in place to inform clinical management of eating disorder patients and one-third of staff (n = 20) who completed the survey reported that they do not have a clear understanding of their professional roles/responsibilities when providing care to a patient with an eating disorder (Table 3).

Compared with their colleagues, dietitians reported a higher level of confidence providing care to eating disorder patients (6.6/10 vs. 6.3/10, Table 3). Dietitians also had a greater awareness of QuEDS guidelines (100% vs. 80%) and are more likely to refer to the guideline than their peers (100% vs. 57%). Nursing staff reported being the most unsure of their professional roles/responsibilities (42%) and reported the lowest level of confidence (6.2) working with ED patients.

Staff expressed positive and challenging aspects of caring for patients with eating disorders and offered suggestions for improving care (Table 4).

**Table 4:** Staff reported barriers, enablers suggestions for improving patient care (in order of most frequently reported)

Enablers	Skilled dietitian, nutrition provision/meal plans, support from dietetic assistants, availability of good care plans, adherence to the QuEDS guideline, multidisciplinary team communication, nursing monitoring, medical monitoring, attitudes of staff, QuEDS support, foodservice
Barriers	Guidelines not consistently followed, lack of knowledge and experience in the team, inadequate psychological management, lack of consistency in care, 1:1 CPO process not followed, multidisciplinary team management, understaffed, lack of meal time support, lack of out of hours service, medically cleared too fast, feeding protocols, poor team communication, lack of awareness of QuEDS guideline among staff
Suggestions for improvement	Education and training for staff, specialised eating disorder team, increased nursing time/availability, local protocol/procedure/guideline to inform treatment, nasogastric feeding commencement protocol, increased specialist support from QuEDS, mandatory training, increased mental health support for patients, care commencement in emergency department, specialised ward, bridle NGT

#### The implementation blueprint

The results of the chart audit (evidence-practice gap identification) and staff survey (perceptions knowledge and suggestions for improvements) as well as facilitated observations were used to develop an implementation plan with the aim to align eating disorder management with best-practice (QuEDS) guidelines [8]. The ERIC compilation was used to select discrete strategies that will be used to build a tailored non-linear multicomponent strategy for implementation (Table 5) [14]. The selected strategies aimed to overcome identified barriers to implementing best-practice care which were identified via the staff survey, informal stakeholder consultation and observations.

The following are key ERIC strategies [14] incorporated in phase one of the project are as follows:

- Assess for readiness and identify barriers and enablers.
- Use an implementation adviser.
- Implementation facilitation (ongoing).
- Audit and feedback.
- Obtain formal commitments.
- Conduct local consensus discussions.
- Develop an implementation blueprint.

**Table 5:** Implementation blueprint outlining proposed strategies to achieve practice change goals according to the Expert Recommendations for Implementing Change compilation [14]

Objective	Activities	ERIC strategy
Communicate evidence-practice gap to highlight areas for improvement	Present and disseminate results of chart audit and staff survey Facilitate discussion	Audit and feedback Conduct educational meetings
Enhance leadership engagement	Communicate findings of audit and feedback to executive and leadership Formalise support to implement QuEDS or local guidelines	Involve executive boards Obtain formal commitments Inform local opinion leaders
Improve communication and MDT approach to care	Formation of stakeholder group including director of nursing, nursing unit managers, senior medical officers, consultant liaison psychiatrists, director of nutrition and dietetics, eating disorder dietitian, emergency department medical officer and service improvement officer. Formalise consensus for improvement activities Include identification and training of champions	Build a coalition Organise implementation teams and team meetings Identify and prepare champions Develop stakeholder interrelationship Conduct local consensus discussions
Reduce time to receiving appropriate meal plan/nutrition provision on medical wards	Local eating disorder management protocol with process for medical teams to commence enteral feeding plan Emergency department flow chart for assessment of eating disorder patients and commencing NG feeds for patients meeting criteria for medical admission Education/in services for medical teams at medical grand rounds	Develop and distribute educational materials Provide ongoing consultations Conduct ongoing training Conduct cyclical small tests of change Revise professional roles
Standardise admission pathways	Work with emergency department to standardise assessment of eating disorder	Assess and redesign workflow
Decrease dietetic time	Local eating disorder management protocol with pathway for early NGT insertion and enteral feed initiation by medical officer where indicated *(develop education materials)*	·Develop educational materials Conduct educational meetings Remind clinicians Revise professional roles
Increase staff confidence & knowledge	Create an eating disorder team with patients admitted to designated medical ward where possible, under consistent team to allow for upskilling of a smaller staff group Ongoing training and reminders of local guideline and eating disorder patient care Include MDT approach to QuEDS external consultations	Create new clinical teams Identify early adopters Capture and share local knowledge Remind clinicians
A greater percentage of patients receiving refeeding on medical wards where indicated	Emergency department flow chart for assessment of eating disorder patients emergency department training Local eating disorder management protocol with clear consistent pathway for medical refeeding	Develop education materials Conduct training Inform local opinion leaders Remind clinicians
Understand impact of changes on eating disorder management	Monitor improvement activities Develop an evaluation plan	Develop and implement tools for quality monitoring Conduct cyclical small tests of change

## Discussion

Anecdotally, eating disorder care at regional and small metro Queensland hospitals has been inconsistent, particularly as many lack a specialist eating disorder service and local protocols or guidelines and specialist staff. The Queensland Eating Disorder Service publish guidelines to enable consistency of care and through the QuEDS service [8] hospitals are also able to access a specialist QuEDS consultation service. However, the publication of guidelines in isolation do not result in practice change [15]. Translating research into practice requires an understanding of the evidence-practice gap, contextual factors that can inhibit or enhance change in order to select strategies that enable service improvement [10]. This audit of inpatient admissions to medical and mental health units over a two-year period was the first step in implementing best-practice guidelines for a small metro hospital in Queensland, Australia.

One of the primary goals of inpatient eating disorder treatment as recommended by the QuEDS guideline is medical stabilisation [8]. Individuals who present to hospital with an eating disorder are at high risk of medical instability [16, 17]. The cause of medical complications can be due to the amount of weight loss, how rapid the weight loss has been and compensatory behaviours (vomiting, laxative abuse, appetite suppressants and/or compulsive exercise) that the patient may be engaging in [2, 4]. 100% of patients admitted to a medical ward in our study met the QuEDS medical admission criteria. 50% of patients admitted directly to the mental health also met at least one criteria for medical admission but no patients required escalation of care. Standardising medical assessment for patients with an eating disorder admitted to the emergency department is a key strategy to optimising eating disorder management in line with best practice.

Nutritional resuscitation, another key treatment recommendation, is necessary to reverse the effects of starvation and achieve medical stability [8]. Delayed provision of adequate nutrition in a malnourished patient may exacerbate medical instability and increase duration of stay [16]. Appropriate provision of nutrition to medically unstable eating disorder patients can reduce refeeding risks and promote rehydration. Commencement of 24hr continuous nasogastric feeding is the recommended method of nutrition provision for patients assessed as medically unstable and is well supported by evidence [8]. Based on our results, commencement of recommended nutrition therapy was delayed by an average of two days and greater delays were seen when patients were admitted over weekends outside of dietetic business hours. This highlighted another key area for improvement to enable any MDT member to commence enteral feeding according to a standard protocol. Historically, the dietitian has been the only health professional to commence enteral feeding and oral feeding plans. Therefore, there is great opportunity to systematise local processes to facilitate early NGT insertion and feeding commencement by medical officers to reduce time to safe feeding commencement.

Only a small number of Australian studies have reported the inpatient management of adult eating disorder patients and improvements to care have been demonstrated following implementation of best-practice guidelines [5, 6]. The implementation of a state-wide service plan at another small, non-specialist, regional Australian hospital, resulted in 100% of patients admitted to a medical ward (n = 9) received NG feeding [5]. Average LOS for this study was 46.4 days (15–123), considerably higher than in the present study of 21.7 days (10.2) [5]. However, weight restoration was also greater, accounting for the increased admission requirement to restore weight per recommendations [5]. 21% of the patients we audited for our study achieved the recommended two BMI band increase prior to discharge.

Another recent Australian study highlighted the advantages of criteria-based anorexia nervosa management, particularly relating to refeeding electrolyte derangement [6]. In terms of age, admission BMI and sex, our patient cohort is not dissimilar to the demographics reported in Braude et al. (2020) [6]. Admission criteria of postural tachycardia, electrolyte derangement, hypoglycaemia and postural blood pressure changes were also similar to previous studies [5, 6]. Our study uniquely reported the number of patients at risk of refeeding, as well as those who experienced refeeding electrolyte derangement during their admission. To reduce the risk of refeeding, a prescribed incremental feeding plan and IV/IM thiamine prior to feeding is recommended. Of the 15 audited patients who were assessed as being at risk of refeeding, less than half (n = 6) were commenced on thiamine prior to oral or NG feeding. Compared with other studies, our patient cohort experienced less refeeding electrolyte derangement, however, this may be indicative of less regular biochemical monitoring rather than lower acuity or severity of the eating disorder.

Understanding barriers and enablers to guideline implementation is an important step in developing strategies to translate research into practice [15]. Our staff survey provided additional insight into the contextual factors influencing change to practice. Notably, many staff perceived eating disorder patients as a challenging patient cohort to care, not dissimilar from previous studies [18–20]. Additionally, staff reported they lacked confidence providing care and approximately a third of staff respondents reported that they do not have a clear understanding of their professional roles/responsibilities when providing care to a patient with an eating disorder. Previous reports demonstrate that that 97% of the health workforce has not received sufficient training in eating disorders to feel confident providing treatment [1]. In the *National Eating Disorders Framework* [1] it was stated that: “a key issue for non-specialist clinicians is knowing that information and support are available at an early stage in planning treatment” (p.62). Additionally, 20% of our staff were unaware of the QuEDS guidelines and just over half of staff reported that they were very likely to refer to it. When asked about suggestions for improvement, staff overwhelmingly recommended local guidelines and resources as well as ED specific training. The staff survey highlighted that well documented implementation strategies or providing education and training, skill development and clear professional roles and responsibilities are likely to improve eating disorder management.

The current study is not without its limitations. It is possible that the retrospective audit did not capture all patient admissions for eating disorders during the study period due to limitations with patient identification through clinical coding. Additionally, the staff survey didn’t differentiate between Mental Health and Medical staff to identify the unique problems/staff perceptions across wards. Finally, we did not involve consumer perceptions and experiences of eating disorder treatment as part of the context assessment due to the sensitive nature of discussing care with this patient group and the level of study resourcing available to the lead dietitian.

## Conclusions

This audit and staff survey highlighted gaps between evidence-based practice and current eating disorder patient care at a small metro Queensland hospital, highlighting opportunities to align care with recommended guidelines. The addition of a staff survey provided an understanding of staff perceptions, knowledge and attitudes to inform areas for improvement including education and clarification on professional roles. The findings of this phase 1 implementation project has allowed for targeted implementation strategies to improve patient care and provides important insights for others looking to improve evidence-based care with the adult eating disorder population.

## Data Availability

Data will be made available upon reasonable request upon contacting the corresponding author.
